# An EEG Tool for Monitoring Patient Engagement during Stroke Rehabilitation: A Feasibility Study

**DOI:** 10.1155/2017/9071568

**Published:** 2017-09-24

**Authors:** Gadi Bartur, Katherin Joubran, Sara Peleg-Shani, Jean-Jacques Vatine, Goded Shahaf

**Affiliations:** ^1^Rehabilitative Psychobiology Laboratory, Reuth Research and Development Institute, Reuth Rehabilitation Hospital, Tel Aviv, Israel; ^2^Department of Rehabilitation, Reuth Rehabilitation Hospital, Tel Aviv, Israel; ^3^Rehabilitation and Motor Control of Walking Laboratory, Department of Physiotherapy, Ben-Gurion University of the Negev, Beersheba, Israel; ^4^Sackler Faculty of Medicine, Tel Aviv University, Tel Aviv, Israel; ^5^BrainMARC Ltd., Yokneam, Israel

## Abstract

**Objective:**

Patient engagement is of major significance in neural rehabilitation. We developed a real-time EEG marker for attention, the Brain Engagement Index (BEI). In this work we investigate the relation between the BEI and temporary functional change during a rehabilitation session.

**Methods:**

First part: 13 unimpaired controls underwent BEI monitoring during motor exercise of varying levels of difficulty. Second part: 18 subacute stroke patients underwent standard motor rehabilitation with and without use of real-time BEI feedback regarding their level of engagement. Single-session temporary functional changes were evaluated based on videos taken before and after training on a given task. Two assessors, blinded to feedback use, assessed the change following single-session treatments.

**Results:**

First part: a relation between difficulty of exercise and BEI was identified. Second part: temporary functional change was associated with BEI level regardless of the use of feedback.

**Conclusions:**

This study provides preliminary evidence that when BEI is higher, the temporary functional change induced by the treatment session is better. Further work is required to expand this preliminary study and to evaluate whether such temporary functional change can be harnessed to improve clinical outcome.

**Clinical Trial Registration:**

Registered with clinicaltrials.gov, unique identifier: NCT02603718 (retrospectively registered 10/14/2015).

## 1. Introduction

Stroke is one of the leading causes for long-term functional impairment worldwide. The mechanisms underlying effective rehabilitation following stroke have been the subject of extensive research. There is growing evidence that rehabilitation is most effective when therapists promote active patient participation in the process and full commitment to it, for example, following stroke [1, 2]. Significantly better outcome was achieved by engaged than by nonengaged patients [3, 4]. The underlying factors that seem to define the basic engagement of the patients include their emotional state and level of cognitive function [1, 5]. Factors that have a transient effect during a given rehabilitation session include the importance of the patient ascribing to the session goal, patient-therapist relations, and the exercises used [6, 7]. Other transient factors contributing to engagement are the match between the degree of exercise difficulty and the patient's current functional level [8], as well as boredom and tiredness [1]. In neuropsychological terms, increased engagement appears to correlate with increased patient attention during exercise [3]. Positive clinical outcomes, such as reduced depression and better cognitive and motor outcome, have been shown to correlate with the amount of effective recruitment of attention [1, 9, 10]. There is neurophysiologic evidence suggesting that enhanced recruitment of attention and engagement increases significantly the activation of brain regions involved in motor rehabilitation [11, 12]. Such increased activation may form compensatory connections to overcome reduced activity in these regions due to neurological disease. Increased activation driven by attention results in greater brain plasticity, which may underlie effective rehabilitation [13].

The practical role of engagement was also demonstrated in the improved clinical outcome achieved with robot-assisted rehabilitation when active patient participation is encouraged, as compared to passive protocols [14]. Nevertheless, we are not aware of any currently established marker for patient engagement or attention and therefore the generation of such marker seems to be of clinical importance.

To date, multielectrode EEG (ElectroEncephaloGraphy) systems have been shown to provide effective markers for attention [15]. But obtaining such markers necessitates a relatively long sampling time (in the range of at least several minutes) [15]. Furthermore, it is too cumbersome and therefore impractical to connect patients to a multielectrode EEG system on a regular basis for rehabilitation sessions. Effective harnessing of EEG-based measures for use in a rehabilitation setting should enable real-time feedback as opposed to feedback after many minutes [16, 17]. A tool that provides therapists with real-time feedback on attention recruitment can serve as an objective basis for real-time adjustments during treatment, which can significantly improve rehabilitation outcome in general and after stroke in particular [18].

Our previous research showed that it is possible to extract effective markers for attention from a single-channel EEG system [19, 20]. Furthermore, we simplified EEG analysis to adjust the extraction of relevant attention-related markers from ongoing EEG, without the need for external cues, on the basis of component template matching of the pattern identified by the averaged event-related potential (ERP). [21]. Template matching is the search in the sampled EEG data for a specific a priori pattern. We follow in this regard a known methodology, which scans the raw EEG data for patterns, which were identified in the averaged ERP signal [22]. Since the template we use is a marker for attention [19] we assume that the matched marker we use in this study (termed BEI, Brain Engagement Index) is also a marker for attention. It should be emphasized that, based on the above, a marker for engagement or for attention would be relevant to almost any poststroke dysfunction regardless of its precise localization. Therefore we chose in this preliminary feasibility study a variable population of patients in terms of functional level and site of injury.

The aim of the present study was to evaluate the applicability of a single-channel EEG marker, the Brain Engagement Index (BEI), during standard motor rehabilitation treatment sessions. In the first part we aimed at evaluating the relation of the BEI and functional performance in control participants, hypothesizing that BEI would peak when the participant is required to perform on a higher level, yet the demands are still not overly difficult. The level of difficulty in this part was set using a robotic training system. In the second part we evaluated the relationship between the BEI level during standard stroke rehabilitation sessions and temporary functional changes induced during these sessions. On the basis of the importance ascribed to brain engagement, as presented above [1–4], we hypothesized that higher BEI will be associated with better temporary functional change. For the sake of evaluating the temporary functional change, induced by the single physiotherapy session, we followed an established method of filming the target movements before and after sessions for evaluation of change by blinded observers [23].

## 2. Methods

The study consisted of two parts. The first part evaluated the effects of exercise difficulty and repetition on the BEI. The evaluation was standardized by using a robotic training system (ArmTutor) and by sampling normally functioning control patients. The second part evaluated the applicability of the BEI to patients undergoing standard, nonrobotic, motor rehabilitation sessions after stroke. Both parts of the study were approved by the Institutional Review Board of Reuth Rehabilitation Hospital, and all the participants signed informed consent forms.

## 3. Participants

### 3.1. First Part

Thirteen unimpaired controls (43–67 years old; 11 females, 2 males), without any neurological or psychiatric deficit, were included. The control participants, a convenience sample, were recruited from the personnel of Reuth Rehabilitation Hospital, Tel Aviv, Israel.

### 3.2. Second Part

Twenty poststroke patients were recruited to the second part of the study. We recruited patients with a full understanding, lack of self-report or documentation of major prestroke neurological and/or psychiatric disorders, and a score of 2–4/5 according to the Kendall muscle grading (https://www.niehs.nih.gov/research/resources/assets/docs/muscle_grading_and_testing_procedures_508.pdf) [24] of the relevant muscle groups. One patient was transferred to another hospital after the first session. For another patient there was disagreement between the blinded observers regarding the degree of session effect. Therefore the sample size of the second part of the study included the remaining 18 patients (39–90 years old, 4 females, 14 males), 1–3 months following stroke.

## 4. Tools

EEG was sampled using the MindWave dry electrode system [25], with one frontal electrode (~Fpz) and one reference electrode on the earlobe, at a sampling rate of 512 Hz. Positioning of the electrode conforms with the goal of monitoring prefrontal activity, which may correlate with attention regardless of the site of lesion [26]. The sampled data were transferred through a wireless connection to the experiment computer, where the BEI was processed.

### 4.1. First Part

We used the ArmTutor [27], a device developed for functional motor rehabilitation of the upper extremity, in this case elbow flexion/extension, with the track task (maintaining a ball within a moving track, with changing slopes). The ArmTutor makes it possible to specify the track width on a continuous scale of ArmTutor-specific units. Four exercise levels of increasing difficulty were selected for this evaluation. The difficulty of the task was mediated by narrowing the track width in even steps from 40 ArmTutor units in level 1 to 10 ArmTutor units in level 4. The ArmTutor is a tool in the arsenal of robotic rehabilitation tools developed by MediTouch Ltd. [28, 29].

### 4.2. Second Part

The second part did not involve the use of designated tools. The study aimed at evaluating a general relation between BEI monitoring and temporary treatment effect for different treatment goals and functional levels. Therefore we included patients and treatments of various types; the objectives of standard physiotherapy for the individual patients are presented in Table 1.

## 5. Experimental Protocol

### 5.1. First Part

Each participant was tested in two blocks in each of the four levels of difficulty (1–4). Each participant performed twice two-minute exercises at levels 1, 2, 3, and 4. A 30-second rest period was given between blocks. The ArmTutor software provided a performance grade (in %) per participant. All tests were conducted in a single session, moving consecutively from one level of difficulty to the next. The control participants were blinded with regard to their BEI level during exercise.

### 5.2. Second Part

**Figure 1:**
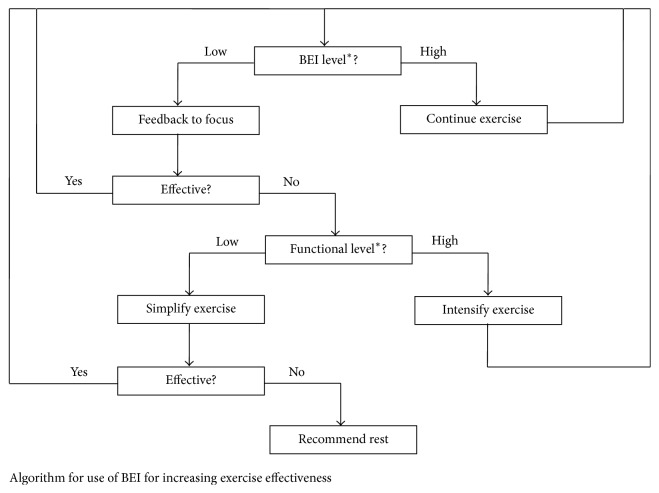
Algorithm for use of the BEI during treatment sessions. If the BEI level was stable, the current exercise continued. When BEI level dropped consistently below average for at least 30 seconds, the patient was first encouraged to concentrate on the exercise, and if this did not help, the therapist evaluated the exercise level. If it was too easy, the therapist intensified the exercise. If it was too difficult, the therapist reduced the intensity of the exercise. If this did not improve the BEI, the therapist suggested rest or used supportive and passive exercises for a few minutes. ^*∗*^Note that both BEI level and functional level are evaluated relatively for each patient.

Each patient underwent an initial evaluation to select a treatment goal for a specific motor function, which matched the patient's functional level. Following this evaluation, each patient completed two treatment sessions. One session involved real-time feedback, with the treating physiotherapist using the BEI; the other session involved BEI monitoring that was not available for the physiotherapist. The order of the two sessions (feedback and no-feedback) was pseudorandomized for each patient through alternate allocation. Altogether, half the patients (9/18) started with the feedback session (FB+) and half (9/18) with the no-feedback session (FB−). During the feedback session, the physiotherapist responded to BEI decreases (of more than 10%) lasting 30 seconds or more by either encouraging the patient to concentrate or by changing the difficulty level of the exercise. A change in difficulty level was based on patient performance: difficulty level was reduced if the exercise seemed to the physiotherapist too difficult for the patient and increased if the exercise seemed too easy.  Figure 1 shows the intervention algorithm used by the therapist. If the BEI level did not decrease, the exercise continued unchanged. Based on these general recommendations, when the BEI level dropped, the physiotherapist did adaptations to the current exercise or switched between exercises. The specific switches in exercise were selected by the physiotherapist, based on his clinical judgment. It should be emphasized that this involvement of judgment by the physiotherapist did not impact the main research question of this study. Note that the main comparison of temporary functional change is between the higher BEI session and the lower BEI session for each patient, regardless of whether feedback was used in the session or not and regardless of the specific exercises selected by the physiotherapist. For some patients the higher BEI session was the session with the feedback, while for others it was the session without the feedback.

Treatment sessions lasted on average 35 minutes. Each session was preceded and followed by a 30-second evaluation period, in which the motor function targeted by the treatment was tested and filmed. The two pairs of evaluation films (FB+ pre/post and FB− pre/post) were evaluated by two physiotherapists, who were blinded to all aspects of the session. The blinded evaluators did not know whether feedback was used and what the BEI level was for the session. Each evaluating physiotherapist was asked to quantify the functional change achieved at the end of each treatment session on a 7-point Likert scale [−3, +3]. The evaluators were instructed to focus their evaluation upon changes in the quality, range, and speed of movement. A positive score indicated improvement and a negative one deterioration. The degree of improvement or deterioration was specified on a scale ranging from 1 (minor) to 3 (major). A score of 0 indicated no significant functional change between the pre- and postsession evaluations. This method of evaluation was suggested by Altschuler et al., 1999. Video-based evaluation was employed in multiple additional studies [30, 31].

## 6. EEG Analysis

**Figure 2:**
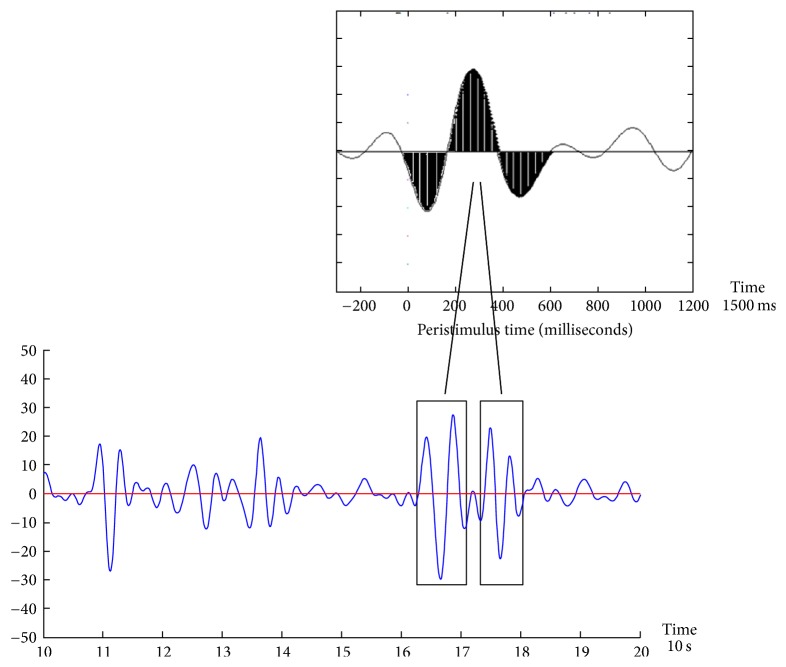
Demonstration of component template matching. The component template is emphasized in black in the top inset. The new sample in the bottom of the figure is scanned with a moving window, following normalization to the [−1,1] range. Whenever a match is found (in black rectangles), it is counted. The BEI is a normalization of this count to the [0,1] range.

The Brain Engagement Index (BEI) was developed by BrainMARC LTD and is available to researchers (see http://brainmarc.com/wp-content/uploads/2015/12/BrainMarc-Brochure.pdf). It is an embodiment of template matching between the averaged ERP signal and the raw EEG sample, which is a prevalent method in advanced EEG analysis, in which a basic template is compared with the sampled signal [21]. The BEI was computed with a moving window of 10 seconds for the period of the preceding 60 seconds. The template was a 1500 milliseconds attention-related averaged ERP delta bandpass activity [19], which was matched with a moving window of the same size in the sampled signal. The matching was performed in real-time every 10 seconds, as follows: (i) the last 60-second sample was divided into segments of 10 seconds; (ii) each segment was filtered in the delta bandpass [1–4 Hz]; (iii) the data points in the filtered segment were normalized to the [−1, +1] range, where −1 denoted the most negative deflection in the filtered segment and +1 the most positive one; (iv) filtering and normalization to [−1, +1] were also performed for the 1500 ms averaged delta ERP wave, shown in Figure 2 (top inset), to generate the template; (v) the normalized sampled segment was scanned by a moving window of 1500 ms; (vi) the averaged distance between the moving window data and both the template and the template opposite (1-template) were computed; (vii) if the averaged distance was less than a threshold of 0.5 from the template (see Figure 2), the count of matches was increased, provided that no other match was found in a previous window, partly overlapping the current one; (viii) if the averaged distance was more than the threshold, the count of no-matches was increased, provided that no other no-match was found in a previous overlapping window; (ix) the BEI is the division of the counts of matches by the no-matches; the maximum BEI value is set to +1, so that the BEI scale has a range of [0,1]; (x) the median of the six 10-second segment BEIs, from the last sampling minute, is taken as the current BEI (as the BEI is calculated every 10 seconds, there is a 50-second overlap in the period analyzed for consecutive BEI values); (xi) for every 1500-millisecond window, we also computed the standard deviation/mean ratio of delta activity. If this ratio was greater than 1, the sampling was likely to be noisy (based on previous analysis) and therefore this 1500-millisecond sample was rejected and not included in the above computation. If multiple (>1) nonoverlapping 1500 millisecond windows were rejected within a given 10-second segment, the entire segment was automatically rejected. At least three nonnoisy 10-second segments were required within the last 1 minute to generate a valid BEI for the entire minute. Otherwise the entire minute was rejected as noisy and no BEI was reported for it. When two consecutive BEI values were not reported (no-value was reported in the monitor graph), the therapist was asked to check and improve the contact of the NeuroSky system with the patient's head, verifying a green connection marker in the application window. Such intervention was required, on average, about 1-2 times in a treatment session. Of potential practical value is the use of dry electrodes below the hairline, Fpz referenced to earlobe. We showed previously the feasibility of extracting significant markers from this region [19]. Obviously sampling from the forehead is always susceptible to noise, especially due to eye movements, but with the use of effective noise rejection method [32] together with adjustment of the device, we were able to obtain 3–6 BEI values in ~90% of the sampling minutes.

## 7. Data Analysis

We used the following indices in the analysis.

### 7.1. First Part

1. Start-of-exercise BEI: we used the first BEI acquired during each exercise block.
2. End-of-exercise BEI: we used the last BEI acquired during each exercise block.

The BEI for each difficulty level was acquired by averaging the 2 blocks of the same difficulty level. In levels 1 and 2 ArmTutor performance grade of all participants was above the MediTouch recommended threshold for good performance. On level 4 all participants showed long drop in performance level below the recommended performance threshold. Level 3 was intermediate with short drops below the recommended performance threshold and thereafter correction to above threshold performance. The recommended performance threshold is used by MediTouch automatically to tune exercise difficulty, but in the current study we deactivated this automatic tuning to maintain constant level difficulties.

### 7.2. Second Part

**Figure 3:**
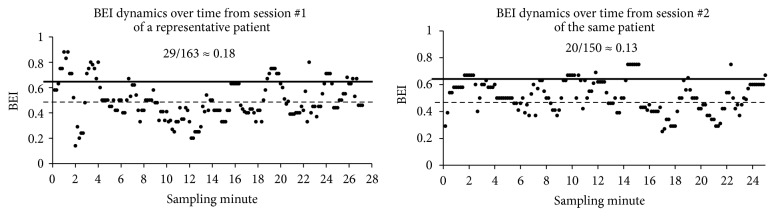
Computation of BEI session. For the sake of clarifying the computation of the BEI session from the basic 10 seconds BEI values, we show an example from one patient of the basic BEI values from the two sessions he underwent. BEI values were computed every 10 seconds for both sessions. The mean BEI (dashed line) and +1 standard deviation (thick line) of all sampled values obtained from* both sessions* were computed. For each session, the number of samples above the mean + 1 standard deviation was counted and was divided by the total number of samples for this session. This value was used as the BEI session, and it is shown for each of the two sessions for demonstration. The same BEI computation session was followed for all patients.

*(1) BEI Session*. We computed BEI values every 10 seconds for both sessions of each patient. Next, we computed the mean BEI and standard deviation of all sampled values from both sessions of each patient. For each session, the number of samples above the mean + one standard deviation was counted and was divided by the total number of samples for the session. This value was used as the entire grade of the session, the BEI session. For the sake of demonstration of the computation of the BEI session, we present Figure 3, which shows the computation of the BEIs session of a representative patient. For each patient, the basic BEI samples are shown as a function of time. The mean BEI for both sessions, taken together, is shown as a dashed line. The mean + one standard deviation is shown as a thick line. The count of BEI samples above this threshold of mean + one standard deviation was divided by the total number of BEI samples from the entire session, to generate the BEI session. Thus if in one of the two sessions of a given patient there was a larger portion of BEI values above threshold than in the other session, this session received a higher BEI session.

*(2) Session Temporary Functional Change Index*. We used the average of the session effect evaluations of the two blinded evaluators as the session outcome index. The evaluation scores given by the two evaluators differed, but the data analysis compared the effect of the two sessions for each participant, and the difference between the two sessions was largely similar between the evaluators; the difference did not exceed 1 point for all patients included in the analysis (one patient, with greater interrater differences, was excluded from data analysis).

## 8. Statistical Analysis

### 8.1. First Part

The comparisons are based on repeated measures ANOVA with Tukey HSD correction and on paired sample *t*-test.

### 8.2. Second Part

The major comparison is based on Wilcoxon-signed ranks test of the session temporary functional change indices. Wilcoxon-signed ranks test is designed for paired-comparison of ordered scales.

A secondary post hoc evaluation was based on chi-square comparison. In this evaluation we compared patients who started with a feedback session, which was followed by a no-feedback session, with patients who started with a no-feedback session, which was followed by a feedback session.

## 9. Results

### 9.1. First Part

**Figure 4:**
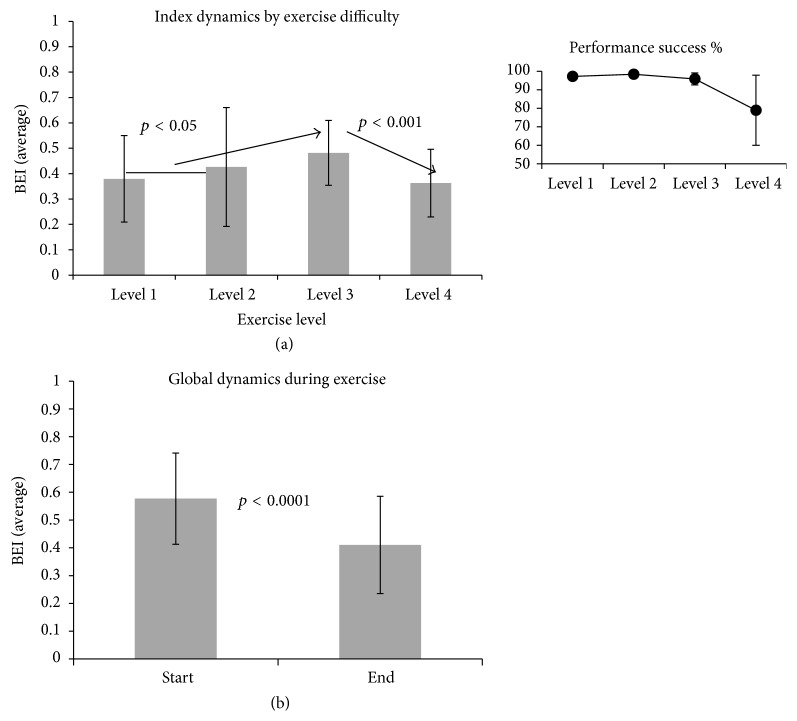
BEI association with exercise difficulty and practice. (a) Dynamics of BEI as a function of exercise difficulty. At each level, the BEI is averaged for both exercises over all participants (±SD). The arrows mark the tendency of BEI change between 3 exercise levels: an average of levels 1 and 2 (owing to functional similarity), level 3, and level 4. The inset shows the percent of success, reported by ArmTutor. The success rate reduced in the 4th level. (b) Dynamics of BEI between start and end of the exercises. The figure shows the decrease of the index between start-of-exercise and end-of-exercise. The start and end columns present averages (±SD) from all 4 levels of exercise (2 exercises in each level) over all 13 controls.

We noted a tendency of increase in average BEI among control participants from levels of exercise difficulty 1 and 2 to level 3. This tendency was reversed in level 4, in which average BEI dropped (Figure 4(a)). To assess the significance of the BEI change, we performed a repeated measures ANOVA between levels with a within-participant factor. We found a significant level effect (*F*(2,42) = 5.99, *p* < 0.01). Follow-up paired sample *t*-tests were conducted separately to compare pairs of difficulty levels and were found to be significant between the first and second levels on the one hand and the third level on the other hand (paired *t*-tests, both with *p* < 0.05) and between the 3rd and 4th levels (decrease) (paired *t*-test, *p* < 0.001). After correction for repeated measures with Tukey HSD, the difference between the 3rd level and 4th level was still significant (*F*(2,11) = 4.29, *p* < 0.01), but the difference between the 1st and 2nd levels and the third level was not significant. The significant drop in BEI between the third and fourth levels was accompanied by reduced performance as reported automatically by the ArmTutor device (Figure 4(a), inset). Participants received an average performance grade of >90% for the first three levels and of ~80% for the fourth level.

Within each level of exercise difficulty there was a decrease in the BEI value from level start to level end (Figure 4(b)). This tendency was statistically significant (paired *t*-test, *p* < 0.0001).

### 9.2. Second Part

**Figure 5:**
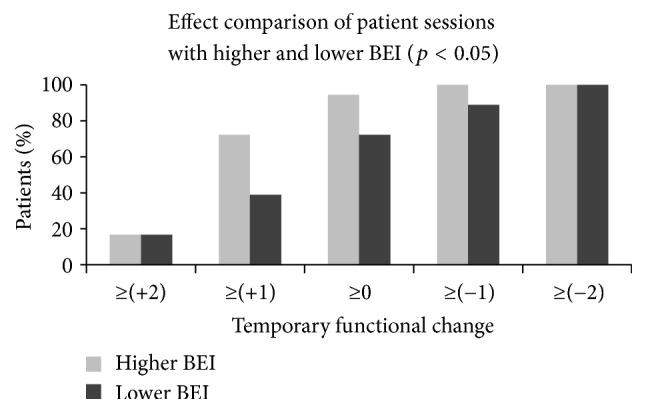
Comparison of the temporary functional change between the sessions in which the BEI was higher for each patient and the sessions in which the BEI was lower for each patient. As each patient participated in two sessions, one of them had by definition higher BEI than the other and the data in the figure aggregates all sessions with higher BEI and all sessions with lower BEI over patients. For some patients the session with higher BEI was the feedback session, while for other patients the session with higher BEI was the no-feedback session. The *y*-axis shows the percentage of patients with a session temporary functional change index above thresholds, which are presented in the *x*-axis. This BEI-based comparison revealed a significant difference in temporary functional change (*p* < 0.05).

The main comparison in this study was between sessions with higher BEI and sessions with lower BEI. As each patient participated in two sessions, one of them had by definition a higher BEI than the other. We grouped the temporary functional changes of the sessions with higher BEI and of the sessions with lower BEI across patients (Figure 5). In the figure the *y*-axis shows the accumulative percentage of patients with a session-induced temporary functional change index above thresholds, which are presented in the *x*-axis. For example, 72% of the sessions with higher BEI of the various patients were rated with temporary functional change ≥+1, but only 39% of the sessions with lower BEI were rated with temporary functional change ≥+1. The difference between sessions with higher BEI and sessions with lower BEI was statistically significant (Wilcoxon-signed ranks test:* Z* ≈ −1.76, *p* < 0.05). It should be noted that this comparison is independent of the physiotherapist involvement in the protocol and exercise selection. The comparison is between higher BEI sessions and lower BEI sessions, regardless of whether feedback was used in the session or not. We further computed the effect size (for categorical variables, [33]) of the higher BEI sessions compared with the lower BEI sessions. This effect size was* d* ≈ 0.71.

**Figure 6:**
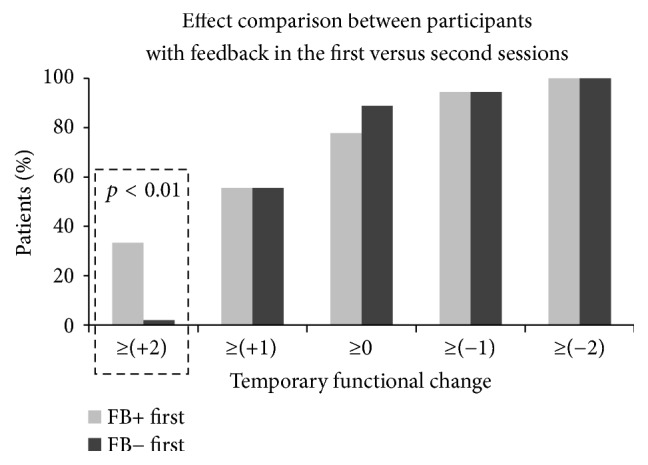
Accumulative histogram comparisons between patients who started with a feedback session and those who started with a no-feedback session. The *y*-axis shows the percentage of sessions with temporary functional change indices above the thresholds, which are presented in the *x*-axis. For participants who started with a feedback+ session, both the first feedback+ and the second feedback− session were included in the count. For participants who started with a feedback− session, both the first feedback− and the second feedback+ session were included in the count. Post hoc analysis of the highest possible temporary functional change (≥+2) revealed a significant difference (*p* < 0.01) between patients who started with feedback in the first session and patients who started with no feedback in the first session. This difference is emphasized with the dashed rectangle.

In a secondary post hoc analysis we compared between patients whose first session was with feedback and patients whose first session was without feedback (Figure 6). For participants who started with a feedback+ session, both first feedback+ and second feedback− sessions were included in the analysis. For participants who started with a feedback− session, both first feedback− and second feedback+ sessions were similarly included in the analysis. The purpose of the comparison was to evaluate whether the use of feedback in the first session had any effect that may have been carried over to the second session. Post hoc statistical analysis showed a significant preference of the maximal temporary functional change (≥+2) for the patients with feedback in the first session in comparison with the patients with no feedback in the first session (*X*^2^(1,36) ≈ 7.20, *p* < 0.01).

## 10. Discussion

The first part of the study suggests there might be a possible association between the BEI and exercise difficulty in a standardized protocol with control participants. BEI at least tended to be lower when the exercise level was easier and it reduced again when exercise level was too difficult and performance was compromised. Thus, BEI may be at its peak when exercise level is challenging, yet not overchallenging. The implication may be that BEI is more related to neurophysiologic processes of sustained attention than with processes of global arousal [34], which may remain high during the phase that is too difficult [35]. It seems possible to distinguish in the EEG signal between sustained attention and alertness markers [36].

Nevertheless, it should be remembered that this is only a preliminary study and more elaborative manipulations of exercise difficulty in various environments are needed in order to establish the relation between the BEI and exercise challenge. If indeed higher BEI will be established further, as related to effective exercise challenge, it may be a useful tool for neural rehabilitation. At times it might be challenging to deduce quickly from good patient performance alone whether a given exercise is sufficiently engaging and the patient works intensively and engagingly or alternatively the exercise is simple for the given patient and does not require intense work. Another required differentiation, in cases of suboptimal functional performance, is between instances in which the patient still works intensively and engagingly and instances in which suboptimal performance is related to reduced engagement. In both cases it is necessary to establish the added value of the BEI for identifying engagement, beyond deduction from performance alone, of both experienced and less experienced therapists.

Supportive evidence for the possible applicability of the BEI as an index for brain engagement comes from its reduction over time within the same level of exercise difficulty. This could be interpreted as habituation secondary to improved dexterity, which develops during practice.

In the second part of the study we evaluated the relation between the BEI of the standard rehabilitative session of poststroke patients and the temporary functional change induced by the session. The assumption was that patient engagement is related to session effectiveness, at least in terms of temporary functional change. As expected, we found a relation between BEI level during the rehabilitative session and the temporary functional change as it was evaluated by skilled blinded observers. The design of the study was not rigorous in terms of interventions employed by the therapist [37]. Furthermore, the target treatment goal was selected individually for each patient, without any attempt at uniformity in treatment goals across patients. Instead it was more naturalistic and the therapist could have selected any intervention he employs in standard rehabilitation sessions. The individualized selection of treatment goals was also chosen because of the expected relation between the relevancy of the treatment goal and the patient's engagement [6]. The main aim was to compare two sessions for each patient. By definition one of such two sessions had a higher BEI than the other and thus it was possible to show that when BEI was higher the temporary functional change was better. The evaluation of temporary functional change had to rely on a short test (so effect will not wear out), which is applicable for multiple functional levels. We used for this purpose the method offered by Altschuler et al. [23]. Similar methods were used also by others [30, 31].

It should be stressed that the subjective involvement of the physiotherapist in the study did not affect the evaluation of the main research question of relation between BEI session and temporary functional change. This is because, all in all, each patient participated in two sessions; in one the BEI was higher than the other. For some patients the higher BEI was in the feedback session, while for others, the higher BEI was in the no-feedback session. But still, as is evident from Figure 5, higher BEI seems to be related to better temporary functional change.

The two treatment sessions for each patient were nevertheless different. In one session the therapist received real-time BEI feedback every 10 seconds and in the other session no feedback was given. The therapist was instructed to change the exercise once the BEI reduced, according to the given algorithm. This enabled post hoc analysis regarding the effect of feedback use on the temporary change in functionality. The seminaturalistic structure of the study and the lack of blinding of the therapist set heavy limitations regarding possible conclusions regarding this secondary question. Nevertheless the therapist was blinded to the type of analysis employed, which compared between patients, who started with a feedback session, and patients who started with no-feedback session. This post hoc analysis revealed that patients, who started with a feedback session, had significantly greater likelihood to demonstrate the highest possible temporary functional change (≥+2). This might mean that the therapist can use the real-time feedback to learn about the best exercises for the specific patient and might further use this information to improve also the second no-feedback session.

This study is only preliminary and of limited sample size and protocol. We believe that the results obtained in such seminaturalistic settings justify further studies with larger samples. It is necessary to establish further the association between BEI and temporary functional change and particularly the use of feedback to improve the temporary functional change. Thereafter it would be of value to evaluate the effect of repetitive use of the BEI in multiple treatment sessions in terms of sustained clinical improvement. This will require a conservative study design of more homogeneous patient populations.

## Figures and Tables

**Table 1:** Demographic and clinical characteristics of patients.

Patient#	Age	Gender	M/P site of lesion^*∗*^	Dysfunction	Target motor function
1	61	M	Lt internal capsule	Hemiparesis (2/5)	Hand to mouth
2	55	M	Lt internal capsule	Hemiparesis (2/5)	Elbow extension
3	48	M	Rt MCA	Hemiparesis (2/5)	Elbow extension
4	52	M	Lt cerebellum	Hemiparesis (4/5)	Stability on one leg
5	70	M	Rt temporooccipital region	Hemiparesis (4/5); hypoesthesia; hemianopsia; hemineglect	Weight shift to the left
6	43	M	Rt internal capsule	Hemiparesis (3/5)	Finger extension
7	61	F	Rt internal capsule	Hemiparesis (3/5)	Finger extension
8	68	M	Lt MCA	Hemiparesis (4/5)	Stability on one leg
9	77	M	Rt MCA	Hemiparesis (4/5)	Finger movement for playing a saxophone
10	69	M	Lt MC	Hemiparesis (4/5)	Sit to stand stability and endurance
11	76	F	Rt MCA	Hemiparesis (4/5)	Preparation to grasp plastic cup
12	66	M	Rt MCA	Hemiparesis (3/5)	Reach and grasp
13	90	M	Lt MCA	Hemiparesis (3/5); aphasia	Manual dexterity: place battery, screw bolt
14	39	M	Lt internal capsule	Hemiparesis (3/5); hypoesthesia	Finger extension
15	57	F	Lt basal ganglia	Hemiparesis (4/5)	Stability of gait
16	72	F	Lt basal ganglia	Hemiparesis (4/5)	Manual dexterity: place battery
17	80	M	Lt superior cerebellum	Hemiparesis (4/5)	Stability of gait
18	67	M	Rt MCA	Hemiparesis (3/5)	Opening of hand

^*∗*^Based on CT scan results and on clinical presentation; MCA: middle cerebral artery.

## Abbreviations

BEI: Brain Engagement Index
ERP: Event-related potential.
